# Effects of different additives on the bacterial community and fermentation mode of whole-plant paper mulberry silage

**DOI:** 10.3389/fmicb.2022.904193

**Published:** 2022-09-08

**Authors:** Changrong Wu, Wentao Sun, Yuan Huang, Sheng Dai, Chao Peng, Yulong Zheng, Chao Chen, Jun Hao

**Affiliations:** Department of Grassland Science, College of Animal Science, Guizhou University, Guiyang, China

**Keywords:** whole-plant paper mulberry, lactic acid bacteria, bacterial community, silage quality, functional prediction

## Abstract

The purpose of this study was to investigate the effects of inoculation with two lactic acid bacteria (LAB) strains (*Lacticaseibacillus rhamnosus* and *Lentilactobacillus buchneri*) and the addition of four corn flour proportions (0, 3, 6, and 9%) in different treatments, on the composition and function of the bacterial community in whole-plant paper mulberry silage. The different treatments promoted *Lactiplantibacillus*, *Lentilactobacillus*, and *Lacticaseibacillus* growth, but the microbial species responsible for fermentation differed among the treatments. High species diversity and various Gram-negative bacteria, such as *Flavitalea* sp., *Pantoea agglomerans*, *Acinetobacter pittii*, *Turicibacter sanguinis*, and *Ralstonia pickettii*, were found in the uninoculated LAB treatments. A beneficial bacterium, *Lactobacillus johnsonii*, was discovered for the first time in whole-plant paper mulberry silage. LAB inoculation simplifies the microbial community structure, and beneficial *Lactobacillus* as a key species aggregates in the inoculated treatment group. However, *L. rhamnosus* inoculation alone may have limited bacteriostatic activity against in whole-plant paper mulberry silage. Compared with silage lacking corn flour, amino sugar and nucleotide sugar metabolism, galactose metabolism, the phosphotransferase system and the pentose phosphate pathway metabolic activity were increased in corn flour-containing silage. Whole-plant paper mulberry can be used as a high-quality silage to provide high-quality feed resources for sustainable ruminant livestock production. Moreover, additive use is necessary for preparing paper mulberry silage.

## Introduction

High elevations, mountainous areas, and severe soil erosion are the primary features of the karst landscape in southwestern China (Chen W. et al., 2021). Ecological restoration and economic development have allowed karst mountain animal husbandry to flourish in this area (Song et al., 2018; Li et al., 2020). However, the explosive development of mountain animal husbandry poses a major challenge to the feed supply. The scarcity of available arable land, feed supply gaps due to changes in the dry and rainy seasons, and a shortage of feed resources are the primary factors restricting the development of mountain animal husbandry (Du et al., 2020). This area is clearly unsuitable for planting and producing large amounts of traditional forages, such as corn, alfalfa, ryegrass and grains and their byproducts. Therefore, the development of new, high-quality feed resources is important.

Paper mulberry (*Broussonetia papyrifera* L.) was originally widely planted in karst mountainous areas due to its strong application prospects in ecological restoration, environmental improvement, water conservation, sand prevention, and sand fixation (Saito et al., 2009; Hu et al., 2020). Because of its high protein content, rich active plant extracts, fast growth, high yield, high quality, multicutting, multiresistance, among other characteristics, there is increasing interest in paper mulberry as a new type of high-quality woody feed. Peng et al. (2019) reported that its yield per hectare was between 45 and 120 tons. Du et al. (2021) reported that twig- and leaf-based dry matter (DM) had a crude protein (CP) content as high as 26% and contained many biologically active compounds, such as flavonoids, lignin, polysaccharides and terpenoids, which may have antibacterial, anti-inflammatory and antioxidant properties for livestock (Han et al., 2016). Tian et al. (2020) found that paper mulberry can also be used as a treatment for various human illnesses and as a source of traditional medicine for animal diseases. Paper mulberry produces a large amount of biomass during the tropical rainy season, and it is impractical to make it into traditional hay under these climatic conditions (Du et al., 2020). Ensiling is an effective way to preserve fresh pasture for a long time (Keshri et al., 2018). Paper mulberry has a high water content, high buffering energy, and low water-soluble carbohydrate (WSC) content, silage fermentation alone results in extensive proteolysis and high butyrate yields, therefore, it is difficult to obtain good quality silage (Ni et al., 2018; Dong et al., 2020). Recent studies have shown that wilting can reduce the water content to inhibit the growth of undesirable bacteria and obtain high-quality silage (Zhang et al., 2019b; Guo et al., 2021). However, during actual large-scale production, prolonged wilting is not only difficult to manage but also necessarily leads to the loss of a large number of nutrients and increases the buffering energy of lactic acid bacteria (LAB; Wang et al., 2019c; Zhang et al., 2019a). Corn flour storage is widespread in this area of China, and it is often used as concentrated feed or an exogenous glycogen additive. In recent years, the use of microbial additives (e.g., LAB) and crop by-products (such as rice bran or corn flour) can effectively change the microbial structure of silage to improve its fermentation quality, thereby improving the feed utilization rate of ruminants (Wang et al., 2019a; Cai et al., 2020; Du et al., 2021; Guo et al., 2021).

However, previous studies mainly used the nutrient-rich tender stems and leaves of paper mulberry as raw materials to prepare silage, focusing on the effects of additives on fermentation products and part of microorganisms, rather than the whole plant (Saito et al., 2009; Han et al., 2016; Du et al., 2020; Guo et al., 2021; He et al., 2021). Among them, these complex microbial structures and interactions have been well characterized thanks to the single-molecule real-time (SMRT) technology that can cover full-length DNA fragments integrated on the PacBio platform. SMRT technology is also widely used to reveal the microbial environment of silage such as whole-plant corn, alfalfa, Napier grass, and sugarcane due to its high throughput, low error rate, and ability to trace microorganisms to the species or even strain level (Cai et al., 2020; Bai J. et al., 2021; Xu D. et al., 2021; Du et al., 2022). To the best of our knowledge, so far, no studies revealing complete microbial information containing whole-plant paper mulberry silage have been reported. More key species and even dominant strains in whole-plant paper mulberry silage are yet to be discovered. In addition, during large-scale production, the branches and edible parts remaining after the removal of the tender leaves and stems are discarded, which is not only a waste of resources but may also create environmental pressure (Eliyahu et al., 2015; Li et al., 2019). Furthermore, the effects of indigenous dominant LAB screened from other silages and low proportions of corn flour on the composition and function of the microbial community of the whole-plant paper mulberry are rarely reported.

To address this gap, this study explored the fermentation characteristics, microbial community composition, and potential functional characteristics of mixed silage with LAB or a low corn flour ratio and whole-plant paper mulberry from economic and practical perspectives. The information provided by this study can serve as a reference for alleviating feed shortages, developing high-quality woody feed, and innovating the economic operation mode of mountain animal husbandry.

## Materials and methods

### Lactic acid bacteria strain screening

From 2016 to 2019, silage microorganisms and feed surface microorganisms were sampled from different representative locations in the center of the karst region in southwest China (Guizhou Province, between 103°36′ ∼ 109°35′E, 24°37′ ∼ 29°13′N, 137–2,900 m above sea level, with a three-season climate) and analyzed. In the end, a total of 130 bacterial species were isolated, of which 49 were identified as LAB. After all strains were isolated and purified, their acid production and growth rate in 24 h were measured on MRS medium (anaerobic at 37°C) (Peng et al., 2021). *Lacticaseibacillus rhamnosus* (LR) strain (BDy3-10), which had the highest acid production rate, and *Lentilactobacillus buchneri* (LB) strain (TSy1-3), which had the fastest growth rate, were selected as the LAB additives in this study (Peng et al., 2021). As shown in the strain characteristics Supplementary Table 1, two strains were Gram-positive and Catalase-negative. Strain BDy3-10 was a homofermentative LAB, and TSy1-3 was a heterofermentative LAB. The two strains grew normally in the range of 20–45°C, but grew weakly at 10 and 50°C. They were able to grow at pH values ranging from 3.5 to 7.0, and grew weakly at pH 3.0 tolerating salt (MRS with 3.0% and 6.5% NaCl concentrations, respectively) which limited their growth. For more detailed information about the LAB used in this research, please refer to our previous work (Peng et al., 2021).

### Whole-plant paper mulberry and corn flour silage preparation

The raw whole-plant paper mulberry materials in this study were collected from Changshun County, Guizhou Province, China on August 27, 2020 (25°43′58.11′′N, 106°24′14.18′′E, 1019 m elevation). The test material was the third harvest of whole-plant hybrid paper mulberry (4–5 harvests can be cut throughout the year). Nine points were randomly selected over a natural planting area (no fertilizers and pesticides were used) of 2 hectares (three field replicates were performed after mixing) for mowing. The cutting height was 15–20 cm above the ground (the height of the whole plant was 150–180 cm). After the plants were wilted for 30 min, a straw cutter was used to chop the material into small pieces measuring 1.5–2.5 cm (Sh-2000, Shanghai Donxe Industrial Co., Ltd., Shanghai, China). Corn flour from traditionally grown corn was passed through a 20-mesh sieve before use. All prepared raw materials were immediately taken to the plantation plant for silage production. Sterilize the accurately calculated corn flour with different ratios (0, 3, 6, and 9%), and prepare with sterilized distilled water when adding, so that the moisture content of the whole-plant mulberry silage is consistent. LAB suspension (1 × 10^8^ cfu/ml, the dosage is 10 ml/kg) and corn flour as additives were used to establish the following treatments on a fresh basis. Mix the chopped whole-plant paper mulberry with the treated corn flour at the ratio of 0, 3, 6, and 9% to make 4 different types of premixes. Each ratio premix makes 12 servings (contains 3 repeats). The treatments for each premix type were NA (No-inoculant, treat with the same volume of sterile distilled water), LR (Inoculation with LR), LB (Inoculation with LB) and M (Combined inoculation of LB and LR). For each treatment, 500 g of sample was mixed with additives and placed in a polyethylene silage bag (30 cm × 40 cm), which was vacuum sealed (Reelanx, Shenzhen, China). In total, 48 bags (4 inoculums × 4 proportions × 3 repeats) were stored at a constant temperature (approximately 25°C) in the laboratory away from light for 60 days before unsealing.

### Chemical and fermentation profile analyses

After 60 days of fermentation, a sample was taken from the middle of the bag to characterize the silage. First, 20 g of fresh sample and silage sample were mixed with 180 ml of distilled water, shaken at low speed, and stored in a refrigerator at 4°C for 24 h for extraction. Next, the extract was filtered through 4 layers of medical gauze, and the pH was measured immediately using a pH meter with a glass electrode (pHS-3C, INESA Scientific Instrument Co., Ltd., Shanghai, China). The remaining extracts were subjected to high-performance liquid chromatography [HPLC; Agilent 1260 Infinity; Column: Agilent TC-C18(2) 250 mm × 4.6 mm 5 μm; oven temperature 50°C; flow rate 0.7 ml/min; SPD 210 nm] to detect lactic acid (LA), acetic acid (AA), propionic acid (PA), butyric acid (BA), and other organic acids. Ammonia nitrogen (NH_3_-N) content was determined by phenol-hypochlorite colorimetric method (Li et al., 2019). Another part of the sample (approximately 200 g) was placed in an oven at 105°C for 30 min and then dried at 65°C to a constant weight to determine DM. The difference in DM before and after silage is DMloss. The concentrations of CP, neutral detergent fiber (NDF), acid detergent fiber (ADF), and WSC were measured on the basis of DM according to the methods of Licitra et al. (1996) and Ke et al. (2017). According to the method provided by Du et al. (2020), the rumen fluid of 3 healthy adult Guanling yellow cattle was collected by nasogastric vacuum tube, the rumen fluid was filtered with gauze, and mixed with buffer solution to determine the IVDMD value.

### Microbial population and bacterial community composition single-molecule real-time analyses

Microbial populations were roughly counted with reference to the plate count method described by Cai et al. (1999) and Wang et al. (2019a). Silage samples (20 g) were added to 180 mL of sterile physiological saline solution (0.85% NaCl), shaken, and then placed in a refrigerator at 4°C for 30 min to mix well, and diluted from 10^–1^ to 10^–8^ in sterile saline. Take 100 μl of each serial dilution and spread it on the plate for 48 h incubation. Enumeration of LAB using deMan, Rogosa, Sharp (MRS) medium agar (Difco Laboratories, Detroit, MI, United States) cultured at 37°C under anaerobic conditions (TEHER Hard Anaerobox, ANX-1; Hirosawa Ltd., Tokyo, Japan). Coliform counts were performed on purplish red bile agar (under aerobic conditions at 30°C). Yeasts and molds were counted on malt extract agar (021110, Huankai Microbial Technology Co., Ltd., Guangzhou, China).

Based on the PacBio sequencing platform (Pacific Biosciences, Menlo Park, CA, United States), single molecule real-time sequencing (SMRT Cell) was used to sequence the marker genes. Raw data were processed using the RS_Readsofinsert.1 protocol in the SMRT Link version 8.0 software.

Database construction and sequencing: Total bacterial genomic DNA was extracted from 48 samples using the TGuide S96 (Tiangen Biotech, Beijing, Co., Ltd.) magnetic bead method genomic kit, A full-length 16S rRNA gene was amplified using primers (27F: 5′-GAGAGTTTGATCCTGGCTCAG-3′; 1492R: 5′-TACCTTGTTACGACTT-3′) and sequenced on the PacBio sequencing platform with single-molecule real-time sequencing (SMRT Cell). The raw circular consensus sequencing data were demultiplexed using lima (v1.17.0^1^). Then, the adapter and primer sequences were removed using Cutadapt 1.9.1 (Martin, 2011).

Information analysis content: Features (operational taxonomic units, OTUs; amplicon sequence variants, ASVs) were classified, Sequences at 97% (default) similarity level were clustered using USEARCH (version 10.0), OTUs were filtered with 0.005% of all sequences sequenced as a threshold and downstream analysis. The taxonomy was assigned to features using SILVA (release 132) as reference. Abundance analysis, alpha diversity, and beta diversity were calculated by QIIME2 (Wang B. et al., 2020). Correlation analysis was performed based on a Spearman’s rank correlation coefficient greater than 0.1 and *P* < 0.05. To analyze functional differences among the treatments, PICRUSt2 software was used to annotate the feature sequence to be predicted (based on the sequenced metagenomic sequence in the KEGG database) and to predict the functional potential of the microbial community during the phylogenetic process based on the correlation between phylogeny and function (Douglas et al., 2019).

### Statistical analyses

The effects of the additives on the fermentation characteristics and chemical composition of whole-plant paper mulberry silage were evaluated using one-way or two-way analyses of variance (ANOVA), with Duncan’s multiple range tests. Alpha diversity indices were compared for significance using Student’s T-test. All statistical procedures were conducted using IBM SPSS 26.0 software. *P* < 0.05 was regarded as a significant difference, and the results are expressed as the average value. Permutation multivariate analysis of variance (PERMANOVA) was performed to compare the differences in microbial communities between treatments. Differentially enriched taxa between four different types of silage (NA, LB, LR, and M) were selected and displayed using LEfSe discriminant analysis, and significant differences were considered by a LDA ≥ 3.0 and *P* < 0.05.

## Results

### Characteristics of raw materials of whole-plant paper mulberry and corn flour before ensiling

The nutritional composition and microbial population of fresh whole-plant paper mulberry and corn flour before ensiling are shown in Table 1. The contents of DM and WSC of the whole-plant paper mulberry were 384.78 g/kg FM and 19.63 g/kg DM, respectively, while those of corn flour were 956.33 g/kg FM and 52.51 g/kg DM, respectively. The CP, NDF, ADF, and ASH contents of whole-plant paper mulberry were 164.71, 490.01, 240.73, and 80.23 g/kg DM, respectively, while those of corn flour were 72.37, 125.17, 25.63, and 6.23 g/kg DM, respectively. In fresh raw material, epiphytic LAB exceeded 5 lg cfu/g, yeast and coliform bacteria counts were 6.17 lg cfu/g FM and 5.40 lg cfu/g FM, respectively, and mold counts were below the detection limit (<10^2^ cfu/g of FM).

**TABLE 1:** Nutrient composition and microbial population of whole-plant paper mulberry and corn flour.

Item	Paper mulberry	Corn flour
Dry matter (g/kg)	384.78	956.33
Crude protein (g/kg DM)	164.71	72.37
Neutral detergent fiber (g/kg DM)	490.01	125.17
Acid detergent fiber (g/kg DM)	240.73	25.63
Crude ash (g/kg DM)	80.23	6.23
Water-soluble carbohydrate (g/kg DM)	19.63	52.51
Lactic acid bacteria (lg_10_ cfu/g FM)	5.23	–
Yeast (lg_10_ cfu/g FM)	6.17	–
Coliform bacteria (lg_10_ cfu/g FM)	5.40	–
Mold (lg_10_ cfu/g FM)	ND	–

DM, dry matter; FM, fresh matter; cfu, colony-forming unit; ND, not detected.

### Chemical and fermentation characteristics of whole-plant paper mulberry silage

The fermentation and chemical characteristics of whole-plant paper mulberry silage are shown in Tables 2, 3. The results showed that the treatments, corn flour ratios, and the interactions of different treatments and corn flour ratios had significant (*P* < 0.01) impacts on organic acids (including LA, PA, and the ratio of LA to AA), DM, CP, and pH. The addition of different corn flour ratios had significant (*P* < 0.001) effects on the contents of DMloss, NDF, ADF, ASH, and WSC. The addition of LAB had no significant effects on the measured parameters except CP (*P* < 0.001). The percentage of corn flour (*P* < 0.001) and its interaction with the LAB treatment (*P* = 0.002) had significant effects on IVDMD (*in vitro* DM digestibility). Although there were some differences in the contents of AA and NH_3_-N, these differences were not significant.

**TABLE 2:** Chemical composition of whole-plant paper mulberry silage after ensiling for 60 days.

		Gradient					*P*-value		
Item	Treatment	0%	3%	6%	9%	SEM	T	*G*	*T* × *G*
pH	NA	4.65aA	4.13b	4.14b	4.04bB	0.017	<0.001	<0.001	<0.001
	LR	4.20aB	4.17ab	4.13b	4.12bA				
	LB	4.20B	4.14	4.14	4.13A				
	M	4.20aB	4.16b	4.18ab	4.15bA				
LA (g/kg DM)	NA	19.20cD	32.74bC	81.69aA	74.33aA	1.965	<0.001	<0.001	<0.001
	LR	63.32bA	53.31cB	76.71aAB	45.89dB				
	LB	42.27cC	60.53bB	74.52aB	46.01cB				
	M	52.16cB	80.49aA	66.48bC	47.84cB				
AA (g/kg DM)	NA	10.47	8.77B	11.32	10.57	0.679	0.671	0.140	0.018
	LR	9.82	7.23B	10.82	10.41				
	LB	9.09b	9.15bB	9.48b	12.39a				
	M	8.99b	12.76aA	9.13ab	10.42ab				
PA (g/kg DM)	NA	7.97cC	27.44bA	30.92aA	7.79cB	0.954	0.003	<0.001	<0.001
	LR	12.28cC	19.73bB	31.89aA	16.22bcA				
	LB	32.15aA	14.59bC	30.91aA	9.84cB				
	M	26.17bB	31.19aA	13.01cB	3.6dC				
LA/AA	NA	1.83cD	3.73bB	7.22aB	7.03aA	0.202	<0.001	<0.001	<0.001
	LR	6.44bA	7.38aA	7.09abB	4.41cBC				
	LB	4.65bC	6.61aA	7.86aA	3.71bC				
	M	5.80bB	6.31bA	7.28aB	4.59cB				
NH_3_-N (g/kg DM)	NA	3.21a	2.82a	2.11bB	2.76a	0.185	0.637	0.524	0.051
	LR	2.48b	2.74ab	3.38aA	2.58ab				
	LB	3.10	3.10	2.52AB	3.09				
	M	3.04	2.65	2.77AB	2.59				

Means with different letters in the same row (a–d) or column (A–B) are significantly different (P < 0.05). NA, samples without inoculants; LB, inoculated with L. buchneri; LR, inoculated with L. rhamnosus; M, inoculated with L. buchneri and L. rhamnosus. Arabic numerals with percentages represent different ratios of corn flour. SEM, standard error of the mean; T, inoculant treatment; G, corn flour ratio; T × G, interaction between the inoculant treatment and corn flour ratio; DM, dry matter; LA, lactic acid; AA, acetic acid; PA, propionic acid; LA/AA, lactic acid to acetic acid ratio; NH_3_-N, ammoniacal nitrogen. Means without letters are not significant.

**TABLE 3:** Fermentation characteristics of whole-plant paper mulberry silage after 60 days of ensiling.

		Gradient					*P*-value		
Item	Treatment	0%	3%	6%	9%	SEM	*T*	*G*	*T* × *G*
DM (g/kg FM)	NA	374.79b	400.94aA	415.11aA	422.82aA	5.62	<0.001	<0.001	<0.001
	LR	371.64b	396.86bA	395.45bA	444.63aA				
	LB	375.70c	404.54bA	401.03bA	437.83aA				
	M	372.30a	137.82bB	139.04bB	128.76cB				
DMloss (g/kg)	NA	9.99a	−16.16b	−30.33b	−38.04b	5.631	0.91	<0.001	0.581
	LR	13.14a	−12.08a	−10.67a	−59.85b				
	LB	9.08a	−19.76b	−16.25b	−53.05c				
	M	12.48a	−12.92b	−15.08b	−48.76c				
CP (g/kg DM)	NA	122.57aC	130.84aB	132.22aB	111.65bB	1.694	<0.001	<0.001	0.002
	LR	139.14aB	133.94aB	140.99aA	123.57bAB				
	LB	147.63aAB	134.16cB	140.11bA	118.48dAB				
	M	146.35aA	137.82bA	139.04bA	128.76cA				
NDF (g/kg DM)	NA	490.72a	478.72a	451.26b	441.29b	3.812	0.336	<0.001	0.636
	LR	490.58a	471.20ab	457.90bc	445.76d				
	LB	502.24a	477.23b	456.00c	452.62c				
	M	499.31a	466.79b	459.18bc	449.49c				
ADF (g/kg DM)	NA	241.68a	231.23ab	220.28b	182.75c	2.746	0.198	<0.001	0.648
	LR	237.08a	232.56a	224.01a	190.21b				
	LB	243.39a	233.50ab	224.96b	196.08c				
	M	242.63a	233.55a	218.61b	182.90c				
ASH (g/kg DM)	NA	83.84a	83.19a	88.49a	56.51bB	2.608	0.341	<0.001	0.087
	LR	73.71b	82.33a	86.47a	67.83bAB				
	LB	85.84ab	82.97ab	87.43a	66.77bAB				
	M	78.33bc	89.70a	84.98ab	73.32cA				
WSC (g/kg DM)	NA	9.00b	23.26abAB	34.61aA	23.84ab	2.439	0.672	<0.001	0.068
	LR	7.97b	28.95aA	17.86abB	30.45a				
	LB	9.56b	23.89aAB	28.75aAB	21.03a				
	M	9.12b	21.89aB	27.06AaB	20.66a				
IVDMD (g/kg)	NA	422.10c	552.50aA	514.17b	570.40aA	11.622	0.124	<0.001	0.002
	LR	467.50	509.73BC	509.73	509.37D				
	LB	454.77b	543.27aAB	513.93a	535.27aC				
	M	457.63c	497.23bC	487.73b	552.97aB				

Means with different letters in the same row (a–d) or column (A–B) are significantly different (P < 0.05). NA, samples without inoculants; LB, inoculated with L. buchneri; LR, inoculated with L. rhamnosus; M, inoculated with L. buchneri and L. rhamnosus. Arabic numerals with percentages represent different ratios of corn flour. SEM, standard error of the mean; T, inoculant treatment; G, corn flour ratio; T × G, interaction between the inoculant treatment and corn flour ratio; DMloss, dry matter loss; CP, crude protein; NDF, neutral detergent fiber; ADF, acid detergent fiber; ASH, crude ash; WSC, water-soluble carbohydrate; IVDMD, in vitro dry matter digestibility. Means without letters are not significant.

With the exception of NA0, the pH was less than 4.2 in all treatments. Except for the M treatment with 6% corn flour, the pH decreased gradually as the ratio of corn flour increased (*P* < 0.05). Compared with NA, LA content increased significantly (*P* < 0.05) in the 0 and 3% corn flour treatments but decreased significantly (*P* < 0.05) in the 6 and 9% corn flour treatments; the LA/AA value was higher (*P* < 0.05) at corn flour ratios of 0 and 3% but lower (*P* < 0.05) at a corn flour ratio of 9%; the CP content increased significantly (*P* < 0.05) in the 6% corn flour, 0% LB, and 0% M treatments. In addition, PA content was highest in the LB0, M0, NA3, M3, NA6, LR6, and LB6 treatments. The DM content increased significantly (*P* < 0.05) as the corn flour ratio increased (*P* < 0.05). DMloss was positive only when the corn flour ratio was 0%. NDF and ADF contents decreased significantly (*P* < 0.05) with increasing ratios of corn flour (*P* < 0.05). Low ASH content was detected in silage with 9% corn flour. The WSC content of the 0% corn flour treatment was significantly lower (*P* < 0.05) than that of the other treatments. IVDMD was generally higher in the treatments with corn flour ratios of 3% and 9%. BA was not detected in any of the treatments in this study (not listed in the table).

### Bacterial community dissimilarities and diversities in whole-plant paper mulberry silage

**FIGURE 1:**
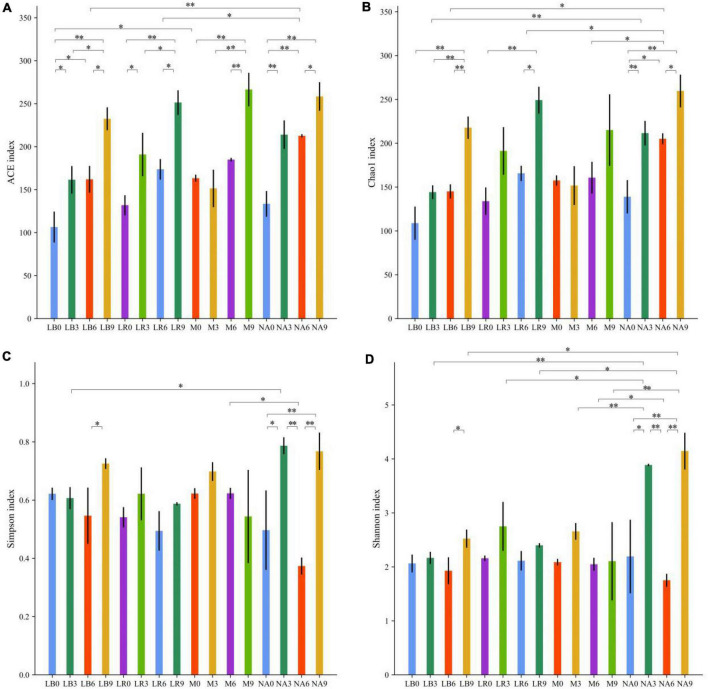
Statistics for bacterial community diversity in silage of whole-plant paper mulberry. **(A)** ACE index; **(B)** Chao 1 index; **(C)** Simpson index; and **(D)** Shannon index. Significance levels for each variable are indicated by *0.01 < *P* ≤ 0.05; ^**^P ≤ 0.01.

**FIGURE 2:**
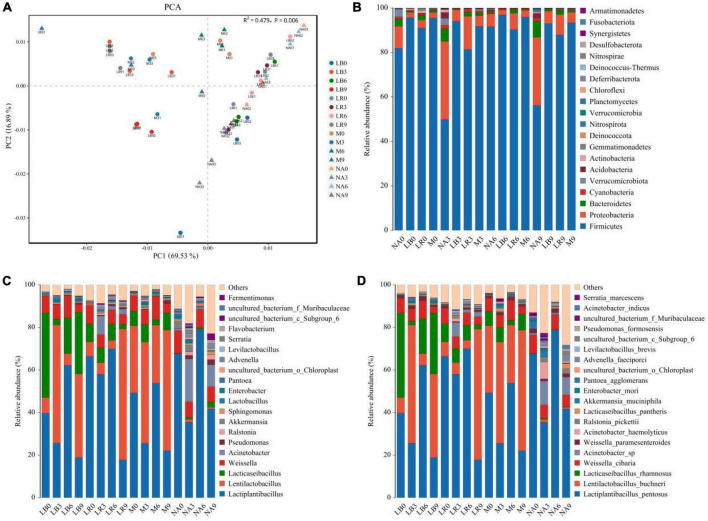
Bacterial community structure in whole-plant paper mulberry silage (*n* = 3). (NA, samples without inoculants; LB, inoculated with *L. buchneri*; LR, inoculated with *L. rhamnosus*; M, inoculated with *L. buchneri* and *L. rhamnosus*. Arabic numerals represent different ratios of corn flour). **(A)** Principal component analysis (PCA) of the bacterial communities in different inoculant treatments (PERMANOVA test with 999 permutations, *P* = 0.001). **(B–D)** Relative abundance of the whole-plant paper mulberry silage bacterial community at the phylum/genus/species level across different inoculant treatments.

Amplification and sequencing of the full-length 16S rRNA of silage bacteria generated an average of 11,955 CCS was retained per sample for downstream analysis. The average effective CCS sequences for analysis after removing chimeras was approximately 92.03%. The sequencing coverage of all samples was higher than 0.99, indicating that sequencing adequately captured the majority of bacteria (Supplementary Table 2). Except for M treatment, addition of corn flour significantly affected the ACE and Chao 1 indices, with higher values in the silage with 9% corn flour (*P* < 0.01), and the Simpson and Shannon indices of some ratios of corn flour, such as NA3 and NA9, were significantly higher than those of the other treatments (*P* < 0.01). There was no general difference in the alpha diversity index with the addition of strains (Figure 1). Principal component analysis (PCA) further revealed differences in the bacterial communities among the treatments at OTU levels. As shown in Figure 2A and Supplementary Table 3, LAB and corn flour and their combined treatment had significant effects on bacterial communities (*R*^2^ = 0.724, *P* = 0.001). Except for LB0, LR0, LR3, and LR6, clearly separation of the treatments with LAB inoculants and treatments with corn flour was observed.

The compositions of the bacterial communities in the paper mulberry silages are shown in Figure 2B (phylum level), Figure 2C (genus level), and Figure 2D (species level). According to the latest *Lactobacillus* taxonomy classification (Zheng et al., 2020), several *Lactobacilli* in this study, such as *Levilactobacillus*, *Lacticaseibacillus*, *Lentilactobacillus* and *Lactiplantibacillus*, have been reclassified into different genera. At the phylum level, *Firmicutes*, *Proteobacteria*, and *Bacteroidetes* were the most important phyla in all silages. The relative abundance of *Proteobacteria* in NA3 and NA9 increased to 35.07 and 30.68%, respectively. Most of the genus-level features originated from *Lactiplantibacillus*, *Lentilactobacillus*, *Lacticaseibacillus* and *Weissella*, followed by *Acinetobacter*, *Pseudomonas*, *Ralstonia*, *Akkermansia*, *Sphingomonas*, *Enterobacter*, and *Pantoea*. The abundance of *Acinetobacter* increased to varying degrees in the NA and LR treatments. After 60 days of fermentation, the dominant bacterial species were *Lactiplantibacillus pentosus*, *L. buchneri*, *L. rhamnosus*, *Weissella cibaria*, *Acinetobacter* sp., *Acinetobacter haemolyticus*, and *Akkermansia muciniphila*. Among these bacteria, *Lactiplantibacillus pentosus*, *L. buchneri*, *L. rhamnosus* and *W. cibaria* accounted for most of the bacterial abundance in all treatments. Inoculation with *L. buchneri* and *L. rhamnosus* increased the abundances of these two bacteria and promoted the growth of *L. pentosus*. Interestingly, the abundance of *L. buchneri* was lower in the *L. buchneri* treatments than in the *L. rhamnosus* treatments, and the abundance of *L. rhamnosus* was lower in the *L. rhamnosus* treatments than in the *L. buchneri* treatments; these two bacteria exhibited opposing abundance trends. *Acinetobacter* sp. was common in the NA treatments and the LR treatments containing corn flour (3, 6, and 9%), and the abundance of *A. muciniphila* decreased as the corn flour ratio increased. The relative abundances of *L. buchneri* and *L. rhamnosus* increased significantly with the addition of a single bacterial agent and corn flour ratios of 3 and 9%.

**FIGURE 3:**
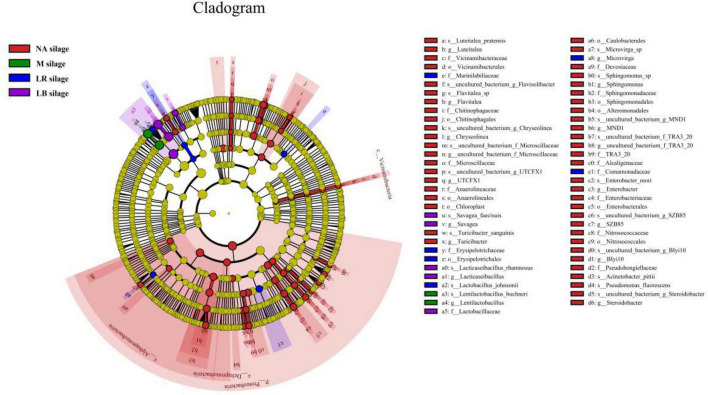
Evolutionary cladogram of different species in whole-plant paper mulberry silage treated with different additives (*n* = 12). NA silage, samples without inoculants or corn flour addition alone; M silage, inoculated with *L*. *buchneri* and *L*. *rhamnosus*; LR silage, inoculated with *L. rhamnosus*; LB silage, inoculated with *L. buchneri*. The circles radiating from the inside to the outside represent the classification levels from phylum to species. Each small circle at a different taxonomic level represents a taxonomy at that level, and the diameter of the small circle is proportional to the relative abundance. Species that are not significantly different are uniformly colored yellow, and other species with differences are colored according to the group with the highest abundance of the species. Different colors represent different groups, and nodes with different colors represent the microbial groups that play an important role in the group represented by the color.

To explain the effects of different corn flour ratios and inoculation on the microbial community of paper mulberry silage, a latent Dirichlet allocation (LDA) effect size (LEfSe) analysis was performed to find markers of differences between the groups (Figure 3 and Supplementary Figure 1). No significant marker microorganisms with an LDA score ≥ 3.0 between treatments with different corn flour percentages were observed. LEfSe identified 67 discriminative features with an LDA score ≥ 3.0 having relative abundances that differed significantly among the LB, LR, M, and NA treatments. At the phylum level, *Proteobacteria* that were unfavorable for fermentation were enriched in the NA treatment. At the genus level, *Flavitalea*, *UTCFX1*, *Turicibacter*, *Sphingomonas*, and *Steroidobacter*, all of which belong to the phylum *Proteobacteria*, were the most different genera in NA-treated silage, while the most different genera in silage inoculated with LB, LR or M were *Lacticaseibacillus*, *Lactobacillus*, or *Lentilactobacillus*, respectively. At the species level, *Flavitalea* sp., *Acinetobacter pittii*, *Turicibacter sanguinis*, *uncultured_bacterium_g_UTCFX1*, *uncultured_bacterium_g_Steroidobacter*, and *uncultured_ bacterium_g_MND1* were identified as differential species in NA-treated silage, while *L. rhamnosus*, *L. johnsonii*, and *L. buchneri* were identified as differential species in silage inoculated with LB, LR, or M different, respectively.

### Distribution and correlation analysis of key bacterial populations in whole-plant paper mulberry silage with different treatments

**FIGURE 4:**
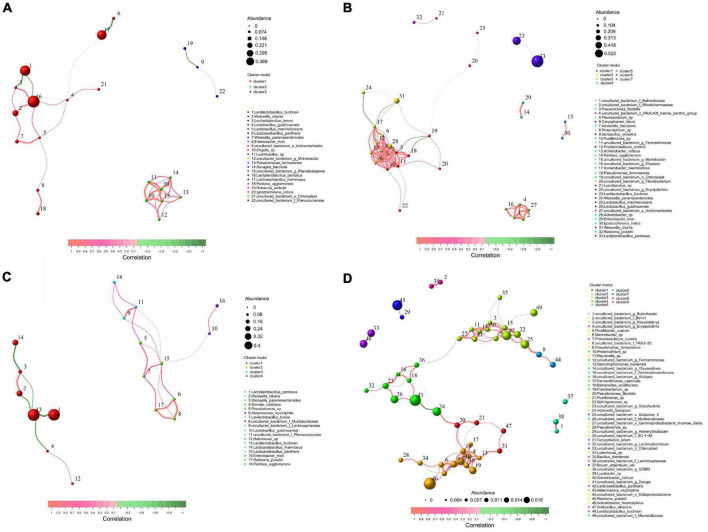
Distribution and correlation analysis of key bacterial populations in whole-plant paper mulberry silage with treatments of **(A)** LB, **(B)** LR, **(C)** M, and **(D)** NA (*n* = 12). LB, inoculated with *L. buchneri*; LR, inoculated with *L. rhamnosus*; M silage, inoculated with *L. buchneri* and *L. rhamnosus*; NA, samples without inoculants or corn flour addition alone.

According to the size of the correlation, LB silage bacteria were divided into 3 key populations, *L. pentosus*, *L. buchneri*, and *L. rhamnosus* were identified as key species. *L. pentosus* was negatively correlated with *L. buchneri*, and positively correlated with *W. cibaria*, *Levilactobacillus brevis*, *L. guizhouensis*, *L. manihotivorans* (*P* < 0.05). *L. rhamnosus* was negatively correlated with *Lacticaseibacillus pantheris* and *L. guizhouensis* (*P* < 0.05) (Figure 4A and Supplementary Data 1). LR silage bacteria were divided into seven key populations, *L. pentosus*, *L. buchneri*, *Pseudomonas formosensis*, *Acinetobacter* sp., *Acinetobacter haemolyticus*, and *Advenella faeciporci* were identified as key species, and *L. pentosus* was negatively correlated with *L. buchneri* (*P* < 0.05) (Figure 4B and Supplementary Data 1). M silage bacteria were divided into four key populations, *L. buchneri*, *L. pentosus*, *L. rhamnosus*, and *L. pantheris* were identified as key species. *L. buchneri* was negatively correlated with *L. pentosus* and *L. rhamnosus* (*P* < 0.05) (Figure 4C and Supplementary Data 1). NA silage bacteria were divided into eight key populations, *L. buchneri*, *L. pantheris*, *R. pickettii*, *A. muciniphila*, and *A. haemolyticus* were identified as key species. *L. pantheris* was positively correlated with 14 bacteria including uncultured *bacterium_c_Subgroup*_6 and *Sphingomonas* sp. (*P* < 0.05); *A. haemolyticus* was positively correlated with 12 bacteria including *Proteiniclasticum ruminis* (*P* < 0.05) (Figure 4D and Supplementary Data 1).

### Functional analysis of the bacterial community in paper mulberry silage

**FIGURE 5:**
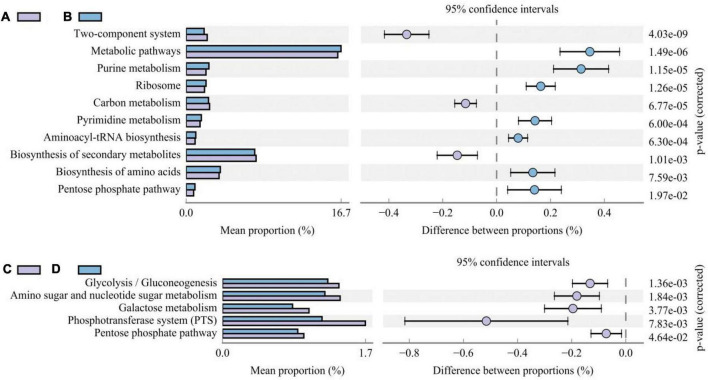
The difference histogram of third-level KEGG metabolism pathways impacted by the absence/presence of LAB **(A/B)** and absence/presence of corn flour **(C/D)**.

Different additive treatments changed the composition of the bacterial community in silage and, accordingly, its functional characteristics. To determine the functional characteristics or metabolic pathways of different bacterial communities, PICRUSt2 was used to predict gene function (based on the third level of the KEGG database). We compared differences in the metabolic pathways between treatments inoculated with and without LAB and those with and without corn flour (Figure 5). Metabolic pathways, purine metabolism, ribosome, pyrimidine metabolism, aminoacyl-tRNA biosynthesis, biosynthesis of amino acids, and the pentose phosphate pathway were higher in LAB-inoculated silage than in uninoculated silage (*P* < 0.05). The metabolic activity of the two-component system, carbon metabolism, and biosynthesis of secondary metabolites were higher in uninoculated silage than in inoculated LAB silage (*P* < 0.05). The metabolic activities of glycolysis/gluconeogenesis, amino sugar and nucleotide sugar metabolism, galactose metabolism, the phosphotransferase system (PTS), and the pentose phosphate pathway were lower in silage with corn flour than in silage without corn flour (*P* < 0.001).

### Correlation analysis of the bacterial community and fermentation characteristics

**FIGURE 6:**
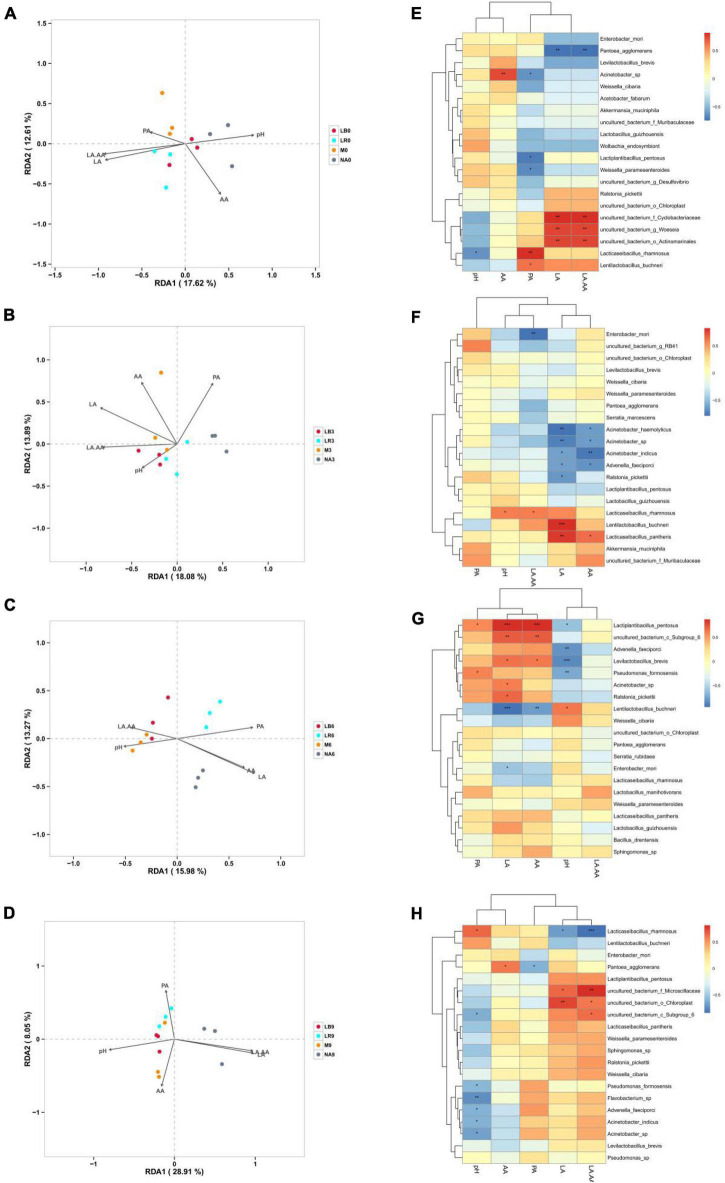
Correlation analysis of the bacterial communities with fermentation characteristics. NA, samples without inoculants; LB, inoculated with *L*. *buchneri*; LR, inoculated with *L*. *rhamnosus*; M, inoculated with *L*. *buchneri* and *L*. *rhamnosus*. Arabic numerals represent different ratios of corn flour. LA, lactic acid; AA, acetic acid; PA, propionic acid; LA/AA, LA and AA ratio; **(A–D)** represent 0, 3, 6, and 9% corn flour ratios, respectively, with RDA. **(E–H)** represent 0, 3, 6, and 9% corn flour ratios, respectively, with Spearman correlation analysis. Correlation coefficient threshold: 0.1, Significance: *P* = 0.05.

In this study, RDA was used to evaluate the influence of fermentation characteristics on the composition of bacterial communities (Figures 6A–D). Shorter arrows for PA in 0% silage, pH in 3% silage, pH and LA/AA in 6% silage and AA and PA in 9% silage, indicating that they have less effect on bacterial communities, while arrows with longer LA had the greatest effect on silage microbial composition across all treatments. At a corn ratio of 0% (Figure 6A), LA and LA/AA were positive correlation with the microbial composition of the treatments inoculated with LR, but negative with that of the uninoculated treatments. At a corn flour ratio of 3% (Figure 6B), LA and LA/AA were positive correlation with the microbial composition of the treatments inoculated with LB and M, but negative with that of the uninoculated treatments. At a corn flour ratio of 6% (Figure 6C), pH was positive correlation with the microbial composition of the treatments inoculated with M, but LA and AA were positive correlation with the microbial composition of uninoculated treatments. At a corn flour ratio of 9% (Figure 6D), pH was positive correlation with the microbial composition of the treatments inoculated with LB and M, but LA and LA/AA were positive correlation with the microbial composition of uninoculated treatments.

The correlations between bacterial species and fermentation characteristics were evaluated using a heatmap (Figures 6E–H). Among all treatments with a 0% corn flour ratio (Figure 6E), *L. rhamnosus* was negatively correlated with pH (*r* = −0.62, *P* < 0.05), and *Acinetobacter* sp. was positively correlated with AA (*r* = 0.74, *P* < 0.01) and negatively correlated with PA. *L. buchneri* and *L. rhamnosus* (*r* = 0.82, *P* < 0.01) were positively correlated with PA. LA and LA/AA were positively correlated with *uncultured_bacterium_f_Cyclobacteriaceae* (*P* < 0.01), *uncultured_bacterium_g_Woeseia* (*P* < 0.01), *uncultured_bacterium_o_Actinomarinales* (*P* < 0.01), *L. rhamnosus* and *L. buchneri* (*P* < 0.05) but negatively correlated with *P. agglomerans* (*P* < 0.01). Among all treatments containing 3% corn flour (Figure 6F), pH was positively correlated with *L. rhamnosus* (*r* = 0.58, *P* < 0.05). Moreover, LA was positively correlated with *L. buchneri* (*r* = 0.83, *P* < 0.001) and *Lacticaseibacillus pantheris* (*r* = 79, *P* < 0.01) but negatively correlated with *Acinetobacter haemolyticus*, *Acinetobacter* sp., *Acinetobacter indicus*, *A. faeciporci*, and *R. pickettii* (*P* < 0.05). AA was negatively correlated with *A. indicus*. Among all treatments with 6% corn flour (Figure 6G), LA and AA were positively correlated with *L. pentosus* and *uncultured_bacterium_c_Subgroup_*6 (*P* < 0.01) and negatively correlated with *L. buchneri* (*P* < 0.01). pH was negatively correlated with *A. faeciporci*, *L. brevis*, and *P. formosensis* (*P* < 0.01). Among all treatments with 9% corn flour (Figure 6H), pH was negatively correlated with *P. formosensis*, *Flavobacterium* sp. (*P* < 0.01), *A. faeciporci*, *A. indicus*, and *Acinetobacter* sp. LA was negatively correlated with *L. buchneri* and *L. rhamnosus* and positively correlated with *uncultured_bacterum_o_Chloroplast* (*P* < 0.01). LA/AA was negatively correlated with *L. rhamnosus* (*P* < 0.001) and positively correlated with *uncultured_bacterum_f_Microsillaceae* (*P* < 0.01), *uncultured_ bacterum_o_Chloroplast*, and *uncultured_bacterium_c_ Subgroup_*6.

## Discussion

The LAB dominate fermentation during the silage process and reduce pH by producing a large amount of LA. The most important indicators of successful silage production are low pH and high LA content (Wang et al., 2019b). The epiphytic LAB count of the whole-plant paper mulberry used in the present study was more than 5.0 lg cfu/g FM, making it favorable for producing high-quality silage (Dong et al., 2020). However, the high abundance of yeast and coliform bacteria in this material can result in poor fermentation quality (Dong et al., 2020) and necessitate the use of additives. As expected, using inoculants and corn flour as an exogenous source of glycogen promoted fermentation. However, increasing the ratio of corn flour did not increase the LA content, possibly because the additional corn flour provided more fermentation substrates not only for LAB but also for microorganisms that are unfavorable to silage fermentation (aerobic bacteria, etc.) (Du et al., 2020). The utilization of fermentation substrates by LAB is limited (Bai et al., 2020), and excess fermentation substrate may allow some unfavorable microorganisms with strong tolerance (such as acid tolerance and facultative anaerobic activity) to multiply or remain latent (Wang et al., 2019a; Yan et al., 2019; Du et al., 2022), which also partially explains the higher microbial diversity in NA3 and NA9 (Figure 2). In this study, the treatment that was neither inoculated with LAB nor supplemented with corn flour (NA) had a high pH and low LA content. The pH values of the other treatments were all less than 4.20, indicating high fermentation quality of the silage (Wang et al., 2019d). Wang B. et al. (2020) reported that excessive production of PA and AA during fermentation leads to low fermentation efficiency or secondary fermentation to produce NH_3_-N. Moreover, Naiara et al. (2019) indicated that heterofermentative LAB produce PA and AA through fermentation to enhance aerobic stability, while homofermentative LAB rapidly produce LA during the early stage of fermentation to reduce pH. Fermentation products are the result of the joint action of the resident microorganisms, and eventually, more PA and a small amount of AA were produced during fermentation because of the different metabolic pathways of the microorganisms (Xu et al., 2019). The LA/AA ratio is an indicator of the fermentation characteristics of silage and reflects, to some extent, the metabolic activity of LAB during the silage period (Xu et al., 2019). When used as a fermentation starter, homofermentative LAB produce a large amount of LA during the early stage of fermentation to quickly lower the pH and promote the growth of heterofermentative LAB (Trabi et al., 2017). We also detected a high LA/AA ratio, low AA content, and an LA/AA ratio greater than 2:1, which may indicate that the different treatments in this study altered the pattern of *Lactobacillus* fermentation in whole-plant paper mulberry silage. Wang S. et al. (2020) attributed the low LA/AA ratio in oat silage to high abundance of the heterofermentative microorganism *L. buchneri*, which enabled the conversion of LA to AA and 1,2-propanediol. This phenomenon was also indicated by the abundance structure of the main microbial species *L. pentosus*, *L. rhamnosus*, and *L. buchneri* in whole-plant paper mulberry silage, which is reported for the first time in the present study (Figure 2D). NH_3_-N content reflects protein degradation in silage, which is primarily due to the synergistic effect of plant enzyme activity, *Clostridium*, and *Enterobacter* (Wang et al., 2019b). In this study, only a small amount of NH_3_-N was produced, with a low pH value (Table 2), as illustrated by the low abundance of *Clostridium* and *Enterobacter* (Figure 2). Although we balanced the moisture content before the experiment, the DM content changed significantly after the addition of corn flour. After 60 days of silage, the DM contents of the 3, 6, and 9% treatments increased (Table 3). Overall, the NDF and ADF contents of the whole-plant paper mulberry silage were relatively high, but these high values did not affect its IVDMD (42 ∼ 57%). Unfortunately, the LAB additives in this study did not result in any obvious degradation of fiber, which may have been due to the relative increase in DM content due to partial water loss during the silage process. A similar result was reported by Bai J. et al. (2021). Compared with NA, the addition of LAB preserved the CP of the whole-plant paper mulberry silage (close to 15%). WSC is used as a fermentation substrate by LAB. An inadequate WSC content will lead to poor silage fermentation quality and even silage failure (Wang et al., 2019a). The addition of exogenous glycogen can increase the fermentation substrate to obtain high-quality whole-plant paper mulberry silage (Du et al., 2021), and the addition of a lower corn flour ratio in this study increased the WSC content and enhanced the fermentation quality.

Silage is accompanied by complex microbial changes, and as ensiling progresses, LAB compete with aerobic bacteria and other undesirable microorganisms, resulting in a decrease in microbial diversity. Ultimately, fermentation is dominated by LAB, and the interaction of microorganisms and their products is one of the factors determining the quality of silage (Cai et al., 2020). The addition of corn meal significantly reduces paper silage alpha diversity (Du et al., 2021). In this study, LAB may have coexisted with some facultative anaerobic or highly tolerant miscellaneous bacteria in the whole-plant paper mulberry silage with a low ratio of corn flour. However, the α-diversity index of the silage treated with a single bacterial agent was relatively low, which effectively inhibited the survival of miscellaneous bacteria. For example, microbial diversity was significantly higher in the NA3 and NA9 treatments than in the NA0 treatment, possibly because the addition of corn flour as an exogenous fermentation substrate simultaneously supplied LAB and harmful microorganisms. Limited utilization of fermentation substrates by LAB (Bai et al., 2020) indirectly enhances the competitiveness of harmful microorganisms. NA0 maintained low α diversity, possibly because the LAB originally present on the paper mulberry had strong competitiveness (Guo et al., 2021). Conversely, an anaerobic environment is favorable for LAB competition, and the small amount of fermentable substrate in NA0 was preferentially utilized by LAB. No additional substrate was available to supply harmful microorganisms in this treatment, effectively inhibiting the activity of harmful bacteria and preventing their growth. Further studies of microbial community structure and composition are necessary. Our previous study showed that *L. rhamnosus* produces acid and grows rapidly (Peng et al., 2021). In this study, no obvious separation of LR combined with a low corn flour ratio (0, 3, and 6%) was observed, which may be due to various factors, such as the high buffer energy and low WSC content of paper mulberry (Dong et al., 2020; Guo et al., 2021). Even the addition of corn flour ratios of 3 and 6% did not make *L. rhamnosus* competitive with other microorganisms in whole-plant paper mulberry silage. The relative abundance of bacterial communities is shown in Figures 2B–D. Interestingly, after 60 days of silage, the inoculated *L. rhamnosus* and *L. buchneri* were not the most dominant species in all treatments, and it is possible that the two exogenous LAB were not as competitive as those originally present in the whole-plant paper mulberry silage. Similar findings were reported by Xu D. et al. (2021) in a whole-crop corn silage study. One possible reason is that the LAB additives used in this study were selected from common pasture silages, such as alfalfa and corn, instead of woody feeds such as paper mulberry. In addition, Guo et al. (2018) found that inoculation of alfalfa silage with *L. buchneri* accelerated the growth of *L. plantarum*. The addition of *L. buchneri* and *L. rhamnosus* in this study may also have promoted the growth of *L. pentosus*. Here, Reclassified overwhelming majority of bacterial genera *Lactiplantibacillus*, *Lentilactobacillus*, *Lacticaseibacillus*, and *Weissella* were first reported in whole-plant paper mulberry silage. The genera *Lactobacillus* and *Weisseria* to which they originally belonged are prevalent in forage crops, and previous studies have shown that the production of LA by obligate heterofermentative bacteria in silage reduces the pH (Cai et al., 1999; Guan et al., 2018). These results may indicate that in the presence of a high ratio of corn flour, the addition of microorganisms promoted or tended to promote heterofermentative metabolic pathways, even with the addition of *L. rhamnosus*. Based on this result, in future work we will evaluate the addition of homofermentative *L. rhamnosus*. The metabolic pathway of this bacterium does not run completely through or dominate the entire fermentation period, and it acts more as a fermentation initiator during the initial stage of fermentation, rapidly producing LA, lowering pH, and promoting the growth of other LAB such as *L. buchneri* and *L. pentosus*. Xu et al. (2019) and Bai J. et al. (2021) reported similar results in corn and alfalfa silage studies. However, this does not mean that the role of *L. rhamnosus* is limited to this, and the function and role of microbial species in the community may not be proportional to their abundance (Power et al., 1996; Banerjee et al., 2018). Guo et al. (2021) also identified *L. rhamnosus* as the most influential bacterial species in naturally fermented paper mulberry silage (after wilting but not treated with additives). *L. rhamnosus* abundance is strongly correlated with LA and CP levels, indicating that inoculation with *L. rhamnosus* inhibits the metabolism of deaminated and decarboxylated amino acids by *Enterobacter* and thus reduces the production of ammonia and biogenic amines, which improves the quality of paper mulberry silage (Guan et al., 2020). *Flavitalea*, *UTCFX1*, *Turicibacter*, *Sphingomonas*, *Steroidobacter*, *Acinetobacter*, *O_Enterobacter*, and other microorganisms that are not conducive to silage fermentation often adhere to silage in different silage periods or in silage with poor quality (Driehuis et al., 2018; Zhang et al., 2019a; Guo et al., 2020). As fermentation progresses, these microorganisms are gradually inactivated or disappear. In this study, these microorganisms were identified as markers in the NA treatment by LEfSe analysis (Weiss et al., 2017), which indicates that NA treatment may have some quality hazards (such as potential pathogenicity and antimicrobial toxin synthesis). However, whether there are worse characteristics requires further research. Furthermore, LEfSe analysis identified *L. rhamnosus* as the most influential microbial species in LB-treated silage in this study. We do not know whether this *L. rhamnosus* originated from our additives or was enriched by natural fermentation. Guo et al. (2021) also identified *L. rhamnosus* as the most influential microbe species in paper mulberry silage after natural fermentation based on LEfSe analysis. However, *L. rhamnosus* was not detected in the early stage of fermentation. The microbial species with the greatest variation of abundance in M-treated silage was *L. buchneri*, indicating that *L. buchneri* maintained a competitive advantage in the combined-treated whole-plant paper mulberry silage. *L. johnsonii*, which does not ferment mannitol or ribose (Zheng et al., 2020), was the microbial species with the greatest variation of abundance in LR-treated silage. *L. johnsonii* was recently shown to significantly enhance the efficacy of immune checkpoint inhibitors in models of cancer and has broad application prospects for the treatment of cancer (microbe-based adjuvant therapy) (Mager et al., 2020). Our paper is the first to report the presence of *L. johnsonii* in whole-plant paper mulberry silage. Future analyses may identify additional microbes present in whole-plant paper mulberry silage.

Numerous studies have shown that there is a complex correlation network in the silage fermentation micro-ecosystem (Bai J. et al., 2021; Du et al., 2021; Sun et al., 2021; Xu H. et al., 2021), while silage with high fermentation quality is characterized by a simplified bacterial-associated structure and aggregation of beneficial microorganisms (Bai C. et al., 2021). The LAB inoculation treatment in this study simplifies the microbial correlation structure and promotes fermentation. Bai J. et al. (2021) and Xu D. et al. (2021) pointed out that key species drive and control the entire fermentation process. However, similar to Xu D. et al. (2021), inoculation of *L. plantarum* in whole corn silage identified some Gram-negative bacteria as key populations, certain bacteria from *Proteobacteria* (such as *P. formosensis*, *Acinetobacter* sp., *A. haemolyticus*, and *A. faeciporci*) were also identified as key species in LR-treated silage. This suggests that *L. plantarum* and *L. rhamnosus*, both homofermentative, may have similar functions during silage fermentation. For example, their bacteriostatic mechanism may be limited to rapid acid production, lowering pH, and inhibiting the growth of acid-sensitive microorganisms, instead of directly producing antibiotics (Zhang et al., 2020), so the inhibitory effect on some facultative acid-resistant microorganisms is not obvious. Minimal population structure and more key LAB species in the silages (LB and M) treated by *L. buchneri* may benefit from the direct antibacterial properties of *L. buchneri*. According to previous studies, homofermentation of *L. pentosus* in paper mulberry silage may inhibit the growth of other LABs (Du et al., 2021). In the present study, a negative correlation between homofermented *L. pentosus* and *L. rhamnosus* and heterofermented *L. buchneri* was observed, which contradicted the expected synergistic effect of combined inoculation of different LAB types. In contrast, the uninoculated (NA) silage had a more complex structure of microbial associations, and many microorganisms that were not conducive to fermentation (such as *R. pickettii*, *A. muciniphila*, and *A. haemolyticus*) were identified as key species. And the key LAB species *L. pantheris* was positively correlated with 14 unfavorable microorganisms (such as *uncultured bacterium*_c_*Subgroup*_6 and *Sphingomonas* sp., etc.), and there was also a positive correlation between unfavorable microorganisms (such as *A. haemolyticus* and *P. ruminis*, etc.). These above represent poor fermentation quality and potential microbial pathogenicity of NA.

In general, most metabolic categories increased over time, which indicates that the metabolic intensity of the microbial community increased as microbial diversity and abundance increased (Qiu et al., 2018). High abundances of beneficial microorganisms were detected in the LAB treatments, which are the primary factors causing these types of metabolism to be active (Chen J. et al., 2021; Du et al., 2021). In addition, the high amino acid biosynthesis activity indicates that adding LAB allows macromolecular proteins present in the silage to be degraded into amino acids or peptides that are easily absorbed by the body (Du et al., 2020). The abundance of secondary biosynthesis was higher in untreated silage than in the treatments inoculated with bacteria (Figure 5), indicating that the absence of added bacteria will result in excessive secondary metabolite production. Specific metabolites must be studied using multiomics methods. Glycolysis/gluconeogenesis, pyruvate metabolism, and amino sugar and nucleotide sugar metabolism were also relatively abundant metabolic pathways in this study (Figure 5); these pathways are second-level KEGG carbohydrate metabolism pathways. Bai J. et al. (2021) and Xu D. et al. (2021) showed that the expression of carbohydrate metabolism pathways is related to the relative abundance of total LAB in the bacterial community. The addition of corn flour and LAB changes the metabolic pathways of microorganisms, which may have a significant impact on the flavor and edibility of animal silage (Du et al., 2020). However, it must be pointed out that the reliability of the above functional predictions is limited, and future studies need to be validated by combining metabolomics or metagenomics.

Studies of silage microbial communities commonly use RDA to evaluate the influence of fermentation parameters on bacterial communities (Wang S. et al., 2020; Bai J. et al., 2021; Guo et al., 2021). In all treatments, the arrows associated with LA and LA/AA were generally longer, indicating that LA and LA/AA had a greater impact on the bacterial communities in all treatments and were determinants of bacterial community formation in whole-plant paper mulberry silage (Bai J. et al., 2021).

The microbial species related to fermentation in silage differed depending on the corn flour ratio. In general, most of the microorganisms that were positively related to LA belonged to the genus *Lactobacillus*. In silage with 0% corn flour (Figure 6E), *P. agglomerans* was extremely negatively correlated with LA and LA/AA. Du et al. (2021) reported that *P. agglomerans* is a rod-shaped Gram-negative bacterium that is occasionally reported to be an opportunistic pathogen in immunocompromised animals and is not conducive to LA production and pH reduction. As the pH decreased, *P. agglomerans* was replaced by *Cyclobacteriaceae*, *Woeseia*, *Actinomarinales*, *L. rhamnosus*, and *L. buchneri*, which were significantly positively correlated with LA and LA/AA. *L. rhamnosus* and *L. buchneri* were positively correlated with PA, consistent with the results of Bai J. et al. (2021) for silage alfalfa. However, Li et al. (2020) reported that if the initial acidification caused by LA fermentation is not effective in preventing the proliferation of *Clostridia* in silage, *Clostridia* fermentation will occur, resulting in PA and butyric acid production or the accumulation of ammonia and amines. He et al. (2020) also reported that rice straw silage with *Moringa oleifera* leaves and propionic acid can inhibit the deamination activity when airtight and exposed to air. This finding shows that the production of PA is regulated by many different types of microorganisms in different forage silages. For *Acinetobacter* sp., a Gram-negative strictly aerobic conditional pathogen, Bai C. et al. (2021) reported that AA metabolism during the aerobic stable phase improves aerobic stability. In the present study, *Acinetobacter* sp. was positively correlated with AA in silage with 0% corn flour, while *Acinetobacter* sp., *A. haemolyticus*, and *A. indicus* were negatively correlated with LA and AA in the 3% corn flour treatment (Figure 6F). A possible explanation is that adding corn flour results in differences in the fermentation substrate such that the rapid growth of LAB produces LA and reduces the pH to inhibit the growth of *Acinetobacter*. By contrast, the growth of *Acinetobacter* was not markedly inhibited in silage without corn flour. Interestingly, *L. buchneri* was negatively correlated with LA and AA in the 6% corn flour treatment (Figure 6G), and *L. rhamnosus* was negatively correlated with LA and LA/AA in the 9% corn flour treatment. In these treatments with higher corn flour ratios, the abundances of *L. buchneri* and *L. rhamnosus* might be reduced, and other highly active LAB might inhibit the production of LA. For example, *Lactiplantibacillus pentosus*, *Subgroup_*6, *L. brevis*, and *R. pickettii* may inhibit acid production by *L. buchneri*, while *uncultured_bacterum_f_Microsillaceae*, *Chloroplast*, and *Subgroup_*6 may inhibit acid production by *L. rhamnosus* (Zhang et al., 2019a; Bai J. et al., 2021; Guo et al., 2021).

## Conclusion

This research provides a variety of options for preparing high-quality silage, especially woody silage. The addition of LAB and/or a low ratio of corn flour changed the bacterial community structure of whole-plant paper mulberry silage in different ways. The microbial species responsible for fermentation differed among the treatments, reducing species diversity, and increases in LA content and reduction of pH resulted in high-quality fermentation. The addition of corn flour as an exogenous source of glycogen had a limited impact on the microbial community, and omission of LAB inoculation or addition of corn flour alone did not completely inhibit the growth of Gram-negative bacteria. LAB inoculation simplifies the microbial community structure, and beneficial *Lactobacillus* as a key species aggregates in the inoculated treatment group. The interactions between strains were the key determinant of microbial community structure. Overall, our results have confirmed the feasibility of inoculating LAB and adding a low proportion of corn flour to produce high-quality silage from whole-plant paper mulberry.

## Data availability statement

The datasets presented in this study can be found in online repositories. The names of the repository/repositories and accession number(s) can be found below: https://www.ncbi.nlm.nih.gov/bioproject/PRJNA854373.

## Author contributions

CRW: investigation, conceptualization, methodology, data curation, formal analysis, visualization, and writing — original draft. WTS: investigation, data curation, and formal analysis. YH: investigation and data curation. SD: investigation and software. CP: investigation and methodology. YLZ: resources and investigation. CC: resources and validation. JH: resources, supervision, conceptualization, writing — review and editing, and funding acquisition. All authors contributed to the article and approved the submitted version.

## Footnotes

1
https://github.com/PacificBiosciences/barcoding
