# Supplementary material for: Effects of different additives on the bacterial community and fermentation mode of whole-plant paper mulberry silage
Source: Front Microbiol. 2022 Sep 8;13:904193. doi: 10.3389/fmicb.2022.904193 (PMC9493322; doi:10.3389/fmicb.2022.904193)
Supplement: Supplementary file 1 [file Data_Sheet_1.ZIP › Data Sheet 1.ZIP!/Data Sheet 1.ZIP!/Table S1, Table S3, Fig S1.DOCX]

***Supplementary Material***

**Effects of different additives on the bacterial community and fermentation mode of whole-plant paper mulberry silage**

**Changrong Wu, Wentao Sun, Yuan Huang, Sheng Dai, Chao Peng, Yulong Zheng, Chao Chen, Jun Hao**

Department of Grassland Science, College of Animal Science, Guizhou University, Guiyang 550025, China

**Corresponding author:**

Dr. Jun Hao

E-mail addresses: jhao@gzu.edu.cn (J. Hao).

**Table S1.** Physiological and biochemical properties of LAB used for inoculation

| Characteristics | BDy3- 10 | TSy1-3 |
| --- | --- | --- |
| Gram stain | + | + |
| Fermentation type | Homo | Hetero |
| Catalase | − | − |
| Growth at pH |  |  |
| 3 | w | w |
| 3.5 | + | + |
| 4 | + | + |
| 4.5 | + | + |
| 5 | + | + |
| 6 | + | + |
| 6.5 | + | + |
| 7 | + | + |
| Growth at temperature |  |  |
| 10 °C | w | w |
| 20 °C | + | + |
| 35 °C | + | + |
| 40 °C | + | + |
| 45 °C | + | + |
| 50 °C | w | w |
| Growth in NaCl |  |  |
| 3% | − | − |
| 6.5% | − | − |

+, positive; −, negative; w, weakly positive; Homo, homofermentative; Hetero, heterofermentative.

**Table S3.** Principal component analysis (PCA) bacterial community difference statistical summary (PERMANOVA test with 999 permutations)

| Characteristics | Num | Df | SumsOfSqs | MeanSqs | F.Model | R^2^ | *P*-value | *P*-adj |
| --- | --- | --- | --- | --- | --- | --- | --- | --- |
| Group | 48 | 15 | 4.461 | 0.2974 | 5.5996 | 0.7241 | 0.001 | 0.001 |

**Figure S1.** LDA value distribution of different species in whole-plant paper mulberry silage treated with different additives. NA silage, samples without inoculants or corn flour addition alone; M silage, inoculated with *L. buchneri* and *L. rhamnosus*; LR silage, inoculated with *L. rhamnosus*; LB silage, inoculated with *L. buchneri*.
